# Multiple Miscarriages Are Associated with the Risk of Ovarian Cancer: Results from the European Prospective Investigation into Cancer and Nutrition

**DOI:** 10.1371/journal.pone.0037141

**Published:** 2012-05-18

**Authors:** Marieke G. M. Braem, N. Charlotte Onland-Moret, Leo J. Schouten, Roy F. P. M. Kruitwagen, Annekatrin Lukanova, Naomi E. Allen, Petra A. Wark, Anne Tjønneland, Louise Hansen, Christina Marie Braüner, Kim Overvad, Françoise Clavel-Chapelon, Nathalie Chabbert-Buffet, Birgit Teucher, Anna Floegel, Heiner Boeing, Antonia Trichopoulou, George Adarakis, Maria Plada, Sabina Rinaldi, Veronika Fedirko, Isabelle Romieu, Valeria Pala, Rocco Galasso, Carlotta Sacerdote, Domenico Palli, Rosario Tumino, H. Bas Bueno-de-Mesquita, Inger Torhild Gram, Oxana Gavrilyuk, Eiliv Lund, Maria-José Sánchez, Catalina Bonet, Maria-Dolores Chirlaque, Nerea Larrañaga, Aurelio Barricarte Gurrea, Jose R. Quirós, Annika Idahl, Nina Ohlson, Eva Lundin, Karin Jirström, Salma Butt, Konstantinos K. Tsilidis, Kay-Tee Khaw, Nick Wareham, Elio Riboli, Rudolf Kaaks, Petra H. M. Peeters

**Affiliations:** 1 Julius Center for Health Sciences and Primary Care, University Medical Center Utrecht, Utrecht, The Netherlands; 2 Department of Epidemiology, GROW - School for Oncology and Developmental Biology, Maastricht University, Maastricht, The Netherlands; 3 Department of Obstetrics and Gynaecology, GROW - School for Oncology and Developmental Biology, Maastricht University Medical Center, Maastricht, The Netherlands; 4 Division of Cancer Epidemiology, German Cancer Research Center, Heidelberg, Germany; 5 Cancer Epidemiology Unit, Nuffield Department of Clinical Medicine, University of Oxford, Oxford, United Kingdom; 6 Department of Epidemiology and Biostatistics, School of Public Health, Imperial College London, St. Mary’s Campus, London, United Kingdom; 7 Institute of Cancer Epidemiology, Danish Cancer Society, Copenhagen, Denmark; 8 Department of Epidemiology, School of Public Health, Aarhus University, Aarhus, Denmark; 9 Inserm, Centre for Research in Epidemiology and Population Health, U1018, Institut Gustave Roussy, Villejuif, France; 10 Gynecology Department Hospital Tenon AP-HP, Paris, France; 11 University Pierre and Marie Curie, Paris, France; 12 Department of Epidemiology, German Institute of Human Nutrition Potsdam-Rehbruecke, Nuthetal, Germany; 13 WHO Collaborating Center for Food and Nutrition Policies, Department of Hygiene, Epidemiology and Medical Statistics, University of Athens Medical School, Athens, Greece; 14 Hellenic Health Foundation, Athens, Greece; 15 Section of Nutrition and Metabolism, Nutritional Epidemiology Group, International Agency for Research on Cancer, Lyon, France; 16 Nutritional Epidemiology Unit, Fondazione IRCCS Istituto Nazionale Tumori, Milano, Italy; 17 Istituto di Ricovero e Cura a Carattere Scientifico - Centro di Riferimento Oncologico di Basilicata, Unit of Clinical Epidemiolgy, Biostatistics and Cancer Registry, Rionero in Vulture, Italy; 18 Center for Cancer Prevention (CPO-Piemonte), Turin, Italy; 19 Human Genetic Foundation, Torino, Italy; 20 Molecular and Nutritional Epidemiology Unit, Cancer Research and Prevention Institute – ISPO, Florence, Italy; 21 Cancer Registry and Histopathology Unit, "Civile - M.P. Arezzo" Hospital, ASP 7 Ragusa, Italy; 22 National Institute for Public Health and the Environment, Bilthoven, The Netherlands; 23 Department of Gastroenterology and Hepatology, University Medical Centre, Utrecht, The Netherlands; 24 Institute of Community Medicine, Faculty of Health Sciences, University of Tromsø, Norway; 25 Escuela Andaluza de Salud Publica, Granada, Spain; 26 Consortium for Biomedical Research in Epidemiology and Public Health (CIBER Epidemiología y Salud Pública-CIBERESP), Barcelona, Spain; 27 Unit of Nutrition, Environment and Cancer, Cancer Epidemiology Research Programme, Catalan Institute of Oncology, Barcelona, Spain; 28 Department of Epidemiology, Murcia Regional Health Authority, Murcia, Spain; 29 Public Health Department of Gipuzkoa, Basque Government, Spain; 30 Navarre Public Health Institute, Pamplona, Spain; 31 Public Health and Health Planning Directorate, Asturias, Spain; 32 Department of Clinical Sciences, Obstetrics and Gynecology, Umeå University, Umeå, Sweden; 33 Department of Public Health and Clinical Medicine, Nutritional research, Umeå University, Umeå, Sweden; 34 Department of Medical Biosciences, Pathology, Umeå University, Umeå, Sweden; 35 Department of Clinical Sciences, Division of Pathology, Lund University, Skåne University Hospital, Lund, Sweden; 36 Department of Surgery, Lund University, Skåne University Hospital, Malmö, Sweden; 37 Department of Hygiene and Epidemiology, University of Ioannina Medical School, Ioannina, Greece; 38 University of Cambridge, Cambridge, United Kingdom; 39 MRC Epidemiology Unit Cambridge, Cambridge, United Kingdom; 40 School of Public Health, Imperial College London, London, United Kingdom; Duke University Medical Center, United States of America

## Abstract

While the risk of ovarian cancer clearly reduces with each full-term pregnancy, the effect of incomplete pregnancies is unclear. We investigated whether incomplete pregnancies (miscarriages and induced abortions) are associated with risk of epithelial ovarian cancer. This observational study was carried out in female participants of the European Prospective Investigation into Cancer and Nutrition (EPIC). A total of 274,442 women were followed from 1992 until 2010. The baseline questionnaire elicited information on miscarriages and induced abortions, reproductive history, and lifestyle-related factors. During a median follow-up of 11.5 years, 1,035 women were diagnosed with incident epithelial ovarian cancer. Despite the lack of an overall association (ever vs. never), risk of ovarian cancer was higher among women with multiple incomplete pregnancies (HR_≥4vs.0_: 1.74, 95% CI: 1.20–2.70; number of cases in this category: n = 23). This association was particularly evident for multiple miscarriages (HR_≥4vs.0_: 1.99, 95% CI: 1.06–3.73; number of cases in this category: n = 10), with no significant association for multiple induced abortions (HR_≥4vs.0_: 1.46, 95% CI: 0.68–3.14; number of cases in this category: n = 7). Our findings suggest that multiple miscarriages are associated with an increased risk of epithelial ovarian cancer, possibly through a shared cluster of etiological factors or a common underlying pathology. These findings should be interpreted with caution as this is the first study to show this association and given the small number of cases in the highest exposure categories.

## Introduction

Increasing parity reduces the risk of ovarian cancer [1]–[3], but the association of incomplete pregnancies with risk remains to be characterized. Within EPIC, an 8% lower ovarian cancer risk per full-term pregnancy has been observed [4]. One could hypothesize that pregnancies that terminate early in gestation reduce ovarian cancer risk to a lesser extent. However, epidemiologic findings remain inconsistent, with some reports showing no associations and others showing either inverse or positive associations (as reviewed in [5]).

Incomplete pregnancies include miscarriages (spontaneous losses) and induced abortions (induced losses because of medical indications or unwanted pregnancy), which have diverse aetiologies and, therefore, their association with ovarian cancer risk might differ. Of particular interest are women who have multiple incomplete pregnancies, as the effect of a single event may be too small to detect and/or the risk may not change in a linear manner with each additional incomplete pregnancy.

The aim of the current analysis was to explore the association of multiple miscarriages and induced abortions with risk of epithelial ovarian cancer in the European Prospective Investigation into Cancer and Nutrition (EPIC).

## Materials and Methods

### Ethics Statement

All participants signed an informed written consent. The present study was approved by the Institutional Review Board of the International Agency for Research on Cancer (IARC) and local institutional review boards in participating centres.

**Table 1:** Baseline Characteristics of Female Participants in the European Prospective Investigation into Cancer (EPIC). *

	Miscarriages^a^							Induced Abortions^b^					
	0		1		2–3		≥4	0	1		2–3		≥4
	n = 176,962		n = 41,800		n = 12,714		n = 1,373	n = 174,538	n = 30,246		n = 11,772		n = 1,808
Age (years), mean (SD)	52.3 (9.1)	52.5 (9.0)		53.1 (9.0)		53.5 (8.8)		52.3 (9.1)	50.4 (8.6)	51.3 (9.0)		53.5 (9.7)	
Height (cm), median (5^th^–95^th^ pct)	161 (151–172)	161 (151–172)		161 (150–172)		160 (150–172)		161 (150–172)	162 (151–173)	161 (150–172)		158 (148–170)	
BMI (kg/m^2^), median (5^th^–95^th^ pct)	24.3 (19.5–33.8)	24.4 (19.5–34.0)		24.7 (19.5–34.7)		25.6 (19.5–35.7)		24.5 (19.5–33.9)	23.8 (19.4–33.3)	24.4 (19.5–34.7)		26.2 (20.3–37.0)	
Menopausal status, n (%)													
Premenopausal	57,024 (32.2)	12,927 (30.9)		3,668 (28.9)		338 (24.6)		57,217 (32.8)	11,498 (38.0)	4,213 (35.8)		543 (30.0)	
Postmenopausal	87,496 (49.4)	21,207 (50.7)		6,716 (52.8)		757 (55.3)		86,928 (49.8)	12,891 (42.6)	5,506 (46.8)		1,020 (56.4)	
Perimenopausal c	32,442 (18.3)	7,666 (18.3)		2,330 (18.3)		278 (20.3)		30,393 (17.4)	5,857 (19.4)	2,053 (17.4)		245 (13.6)	
Age at menopause (years), mean (SD)	48.9 (5.0)	48.8 (5.1)		48.7 (5.3)		47.4 (5.7)		48.8 (5.1)	49.0 (5.0)	49.0 (4.9)		48.5 (5.3)	
Ever had a full–term pregnancy, n (%)	170,748 (97.3)	39,435 (95.8)		11,846 (95.1)		1,220 (91.7)		171,234 (98.7)	27,125 (92.3)	10,759 (93.7)		1,711 (95.9)	
Number of full-term pregnancies (n), median (5^th^ –95^th^ pct) d	2 (1–4)	2 (1–4)		2 (1–5)		2 (1–5)		2 (1–4)	2 (1–4)	2 (1–4)		2 (1–5)	
Ever used oral contraceptives, n (%)	102,285 (58.0)	24,349 (58.5)		7,031 (55.5)		736 (53.9)		99,285 (57.1)	20,594 (68.3)	6,787 (57.9)		701 (38.9)	
Duration of oral contraceptive use (years), median (5^th^ –95^th^ pct) e	6.0 (1.0–25.0)	5.0 (1.0–25.0)		4.0 (1.0–25.0)		3.0 (1.0–25.0)		5.0 (1.0–25.0)	6.0 (1.0–25.0)	5.0 (1.0–25.0)		3.0 (1.0–23.0)	
Ever used hormone therapy, n (%)	42,696 (25.9)	10,664 (27.2)		3,219 (27.0)		356 (27.9)		42,354 (25.2)	7,920 (27.8)	2,829 (25.2)		336 (19.0)	
Duration of hormone therapy (years), median (5^th^–95^th^ pct) e	2.2 (0.3–11.8)	2.3 (0.3–12.0)		2.3 (0.3–12.7)		2.1 (0.3–13.0)		2.2 (0.3–11.1)	2.3 (0.3–12.0)	2.3 (0.3–12.0)		2.1 (0.2–13.0)	

a Data on miscarriages were missing for Bilthoven (the Netherlands), Umea (Sweden), and Norway.

b Data on induced abortions were missing for Bilthoven (the Netherlands), Sweden, and Norway.

c Women who were between 46 and 55 years of age and who had missing or incomplete questionnaire data for menopausal status.

d Among parous women only.

e Among users only.

* Percentages may not add up to 100% because of missing values.

Abbreviations: BMI, body mass index; n, number; pct, percentile; SD, standard deviation.

### Study Population

EPIC is a prospective cohort study initiated in 1992 in 10 European countries: Denmark, France, Germany, Greece, Italy, the Netherlands, Norway, Spain, Sweden, and the United Kingdom. Between 1992 and 2000, a total of 519,978 men and women were recruited. Of these, most were aged 25–70 years and recruited from the general population. Exceptions were the Oxford cohort, UK (based on vegetarian volunteers and healthy eaters), the Utrecht cohort, The Netherlands (based on women attending breast cancer screening), the French cohort (based on female members of the health insurance for state school employees), and components of the Italian and Spanish cohorts (members of local blood donor associations). A full description of the study design and cohort has been published elsewhere [6], [7].

Our analysis is based on data from all 274,442 female participants after *a priori* exclusion of women with prevalent malignancy (n = 19,707) or bilateral oophorectomy (n = 10,500) at baseline, incomplete follow-up (n = 2,209), missing lifestyle data (n = 526), and women from centres with missing information on miscarriages and induced abortions (i.e., Bilthoven, Umea, and Norway). For the analysis on induced abortions, we additionally excluded women from Malmo as information on induced abortions was not available for this centre.

**Table 2:** Incomplete Pregnancies and the Risk of Ovarian Cancer in the European Prospective Investigation into Cancer (EPIC), 1992–2010.

			Model 1^a^	Model 2^b^
	n cases	PY	HR (95% CI)	HR (95% CI)
**Incomplete pregnancies** c				
Never	492	1,456,074	Reference	Reference
Ever	298	936,943	1.00 (0.86–1.16)	1.02 (0.88–1.19)
Number of incomplete pregnancies d				
0 (reference)	488	1,446,005	Reference	Reference
1	172	622,724	0.91 (0.76–1.08)	0.92 (0.77–1.11)
2–3	101	301,724	1.11 (0.89–1.38)	1.15 (0.92–1.43)
≥4	23	46,927	1.74 (1.13–2.67)	1.74 (1.20–2.70)
**Miscarriages**				
Never	665	1,949,976	Reference	Reference
Ever	206	622,878	0.96 (0.82–1.13)	0.97 (0.83–1.14)
Number of miscarriages d				
0 (reference)	665	1,949,976	Reference	Reference
1	136	460,243	0.86 (0.72–1.04)	0.86 (0.71–1.05)
2–3	58	140,525	1.20 (0.91–1.57)	1.23 (0.93–1.61)
≥4	10	15,132	1.94 (1.03–3.63)	1.99 (1.06–3.73)
**Induced abortions**				
Never	646	1,910,876	Reference	Reference
Ever	140	466,700	1.01 (0.83–1.22)	1.04 (0.86–1.27)
Number of induced abortions d				
0 (reference)	646	1,910,876	Reference	Reference
1	85	321,772	0.88 (0.70–1.11)	0.92 (0.73–1.16)
2–3	47	123,316	1.32 (0.97–1.80)	1.32 (0.97–1.81)
≥4	7	18,269	1.40 (0.65–3.01)	1.46 (0.68–3.14)

a Stratified for centre and age.

b Adjusted for parity (number of full term pregnancies) and oral contraceptive use (duration of use), body mass index (continuous), menopausal status (pre-, peri- or postmenopausal), educational level (none, primary school, technical/professional school, secondary school, university), and age at menarche (years, continuous).

c Defined as either having had a miscarriage or an induced abortion.

d Number of incomplete pregnancies is missing for 2 cases. Number of miscarriages is missing for 2 cases. Number of induced abortions is missing for 1 case.

Abbreviations: CI, Confidence Interval; HR, Hazard Ratio, PY, person-years.

### Data Collection

Eligible subjects who decided to participate signed an informed consent form and completed diet and lifestyle questionnaires, which were either interviewer-administered (Naples, Ragusa, Spain and Greece) or mailed self-administered (all other centres). In most countries, participants were invited to a centre for blood collection and anthropometric measurements. Through the baseline questionnaire, women were asked whether they had ever had a miscarriage or an induced abortion, and if so, how many miscarriages and/or induced abortions they have had, as well as the age at first and last abortion. The structure and availability of the questions varied by center. For that reason, all variables were standardized centrally. If a participant recorded either a number of miscarriages/induced abortions or their age at a miscarriage, then, it was assumed that they had experienced a miscarriage. Number of miscarriages/induced abortions have been transformed into 1, 2, 3, 4, and 5 or more. Questions on miscarriages and induced abortions were missing for Bilthoven, Umea, and Norway, while, questions on abortions only were missing for Malmo. Therefore, these centres have been excluded from the specific analyses on miscarriages and/or abortions."

### Determination of Menopausal Status

Women were considered as premenopausal at baseline when they reported having had regular menses over the past 12 months or if they were less than 46 years of age. Women were considered postmenopausal when they reported not having had any menses over the past 12 months, or when they were older than 55 years. Women who were between 46 and 55 years of age and who had missing or incomplete questionnaire data for menopausal status were classified as perimenopausal/unknown.

### Follow-up

Participants were followed up from study entry and until diagnosis of any cancer (except non-melanoma skin cancer), death, emigration, or until the end of the follow-up period, whichever occurred first. Incident epithelial ovarian cancer was defined as ovarian, fallopian tube and primary peritoneal cancer (ICD-O-2 codes C56.9, C57.0, and C48, respectively). Incident cancer cases were identified through either population-based cancer registries or a combination of methods including health insurance records, cancer and pathology registries, and active follow-up through study participants and their next-of-kin. Mortality data were also obtained from either the cancer registry or mortality registries at the regional or national level. The end of the follow-up period was different for different centres and ranged between December 2004 and June 2010.

### Data Analysis

Person-years at risk were calculated from the start of the study until censoring at the date of diagnosis of any ovarian cancer, death, emigration, other loss to follow-up or the date at which follow-up ended, defined as the last date at which follow-up data were judged to be complete or the last date of contact in the centers that used active follow-up.

Hazard ratios (HR) and 95% confidence intervals (CI) were estimated using Cox proportional hazards models with age as the underlying time variable and stratified by centre and age. Multivariate models were adjusted for parity (number of full-term pregnancies), duration of oral contraceptive use (continuous), body mass index (continuous), menopausal status (pre-, peri-, or postmenopausal), education (none, primary school, technical/professional school, secondary school, university), and age at menarche (continuous). Missing covariate values were imputed using mean substitution [8].

We performed a sensitivity analysis restricted to postmenopausal women as these women had complete information on number of miscarriages and induced abortions since they were at the end of their fertile life. In this sensitivity analysis we additionally adjusted for age at menopause (continuous) and duration of hormone therapy (continuous).

An additional sensitivity analysis was performed by excluding the first 5 years of follow-up.

All statistical analyses were carried out using SAS 9.2 (SAS Institute Inc, Cary, NC).

### Results

During a median follow-up of 11.5 years, 1,035 women were diagnosed with incident epithelial ovarian cancer (92.9% ovarian, 3.6% fallopian tube, and 3.5% peritoneal cancers).

Compared with women who never had a miscarriage, women with multiple miscarriages (≥2) were slightly older, had higher body mass index, were more likely to be postmenopausal, less likely to have had a full-term pregnancy, less likely to have used oral contraceptives, and more likely to have used hormonal therapy (Table 1). These differences were larger for women with four or more miscarriages. Compared with women who had never had an induced abortion, women who had had 4 or more induced abortions were slightly older, had a lower body mass index, were more likely to be postmenopausal, less likely to have used oral contraceptives, and more likely to have used hormonal therapy (Table 1).

We observed no association between ever having had an incomplete pregnancy or having had one incomplete pregnancy and risk of ovarian cancer, as compared with never having had an incomplete pregnancy (Table 2). However, women with 4 or more incomplete pregnancies had a significantly higher risk (HR_≥4vs.0_: 1.74, 95% CI: 1.20–2.70; number of cases in this category: n = 23). When the number of incomplete pregnancies was divided into miscarriages and induced abortions, women with a history of ≥4 miscarriages had a statistically significant 2-fold increased risk (HR: 1.99, 95% CI: 1.06–3.73; number of cases in this category: n = 10), although this estimate is based on a small number of cases. No association was observed for women who had only one miscarriage. Results were stronger among women who were postmenopausal at baseline (HR_≥4vs.0_: 2.35, 95% CI: 1.16–4.77; number of cases in this category: n = 8). No substantial differences were observed when restricting analyses to ever-pregnant women and when stratifying by age at first pregnancy (<30 vs. ≥30 years; results not shown). To prevent reverse causation we performed an additional sensitivity analysis (all menopausal states included), for which we excluded the first 5 years of follow-up (9,343 women excluded). This resulted in a stronger association (HR_≥4vs.0_ 2.25, 95% CI: 1.06–4.79; number of cases in this category: n = 7).

Multiple induced abortions were not significantly associated with epithelial ovarian cancer risk (Table 2; HR_≥4vs.0_: 1.46, 95% CI: 0.68–3.14; number of cases in this category: n = 7).

## Discussion

In this large prospective cohort study, we observed a 2-fold increased risk of epithelial ovarian cancer among women with 4 of more miscarriages.

This is the first prospective study that investigated the association of multiple miscarriages with ovarian cancer. Most case-control studies only investigated ever versus never had a miscarriage and did not observe an association [5]. Only one study investigated ≥3 miscarriages and none of the studies investigated ≥4 miscarriages [5]. Moreover, these studies were generally underpowered. The results of our study suggest that an association may be present only for women with multiple miscarriages, who are likely to have a more pronounced cluster of etiological factors leading to recurrent events. As the etiology of recurrent miscarriage itself is not fully understood, it is difficult to argue about plausible mechanisms underlying the observed association in this study. Women who experience multiple miscarriages may have an underlying pathology which predisposes them to ovarian cancer as well. A possible mechanism underlying this association might be an endocrinopathy, such as luteal phase deficiency, which is characterized by inadequate corpus luteum progesterone production resulting in progesterone deficiency [9]–[11]. Progesterone is thought to play a major protective role in ovarian cancer aetiology [12], [13]. Factors related to a lack of progesterone have been consistently shown to increase ovarian cancer risk [12]–[13]. However, the incidence of luteal phase deficiency in recurrent miscarriage is estimated to be only 10–28% [10], thus this would only explain part of the association. It is important to note that all possible mechanisms discussed here are speculative and further research is needed to fully understand this association.

In line with most studies we observed no overall association between induced abortions and ovarian cancer risk, whereas two studies reported a reduced risk [5]. However, our result should be interpreted with care for several reasons. It has been shown that induced abortions are often under- or misreported [14]–[16], and it could be that women who underwent surgical curettage for miscarriage or other medical indications misreported this miscarriage as an induced abortion. On the other hand, in many countries induced abortion is illegal and this may also lead to under-reporting.

Our results suggest that miscarriages and induced abortions may have different effects on ovarian cancer risk and therefore examining the association between incomplete pregnancies and risk of ovarian cancer may obscure any real association.

A major strength of this large cohort study is the prospective design, with detailed exposure and covariate assessment prior to diagnosis. A limitation of our study is the relatively small number of cases in the top categories of incomplete pregnancies. In addition, we do not know in which week of the pregnancy the miscarriage or induced abortion occurred. Miscarriages most frequently occur during the 6th to 12th week of pregnancy and our results, therefore, are most likely driven by these early pregnancies. We have not been able to review medical charts to confirm a women’s self-report of miscarriage/induced abortion. However, underreporting seems more likely than over-reporting. If we indeed have missed some miscarriages or induced abortions, this would cause non-differential misclassification since data on pregnancy loss were collected prior to cancer diagnosis. The result would be an attenuation of the risk estimates. An additional limitation of our study is that information about pregnancy loss was collected at baseline only. Among premenopausal women this information might therefore be incomplete, which again may lead to an attenuation of the association. Among postmenopausal women, for whom these data were complete, indeed, we observed a stronger association between multiple miscarriages and ovarian cancer risk.

In conclusion, our findings suggest that multiple miscarriages are associated with an increased risk of epithelial ovarian cancer. As this is the first prospective study that observed this association, more studies are needed to confirm or dispute our findings.
